# Truncation of Pik3r1 causes severe insulin resistance uncoupled from obesity and dyslipidaemia by increased energy expenditure

**DOI:** 10.1016/j.molmet.2020.101020

**Published:** 2020-05-19

**Authors:** Albert Kwok, Ilona Zvetkova, Sam Virtue, Ineke Luijten, Isabel Huang-Doran, Patsy Tomlinson, David A. Bulger, James West, Steven Murfitt, Julian Griffin, Rafeah Alam, Daniel Hart, Rachel Knox, Peter Voshol, Antonio Vidal-Puig, Jørgen Jensen, Stephen O'Rahilly, Robert K. Semple

**Affiliations:** 1 The University of Cambridge Metabolic Research Laboratories, Wellcome Trust-MRC Institute of Metabolic Science, Cambridge, UK; 2 MRC Metabolic Diseases Unit, Wellcome Trust-MRC Institute of Metabolic Science, Cambridge, UK; 3 Centre for Cardiovascular Science, University of Edinburgh, 47 Little France Crescent, Edinburgh, UK; 4 Department of Biochemistry and Cambridge Systems Biology Centre, University of Cambridge, Cambridge, UK; 5 Laboratory of Lymphocyte Signalling and Development, The Babraham Institute, Cambridge, UK; 6 Louis Bolk Institute, Kosterijland 3-5, NL-3981 AJ, Bunnik, the Netherlands; 7 Department of Physical Performance, Norwegian School of Sport Sciences, P.O. Box 4014, Ulleval Stadion, 0806 Oslo, Norway; 8 Biomolecular Medicine, Division of Systems Medicine, Department of Metabolism, Digestion and Reproduction, Medicine, Imperial College London, The Sir Alexander Fleming Building, London, UK

**Keywords:** Insulin resistance, Diabetes, Lipotoxicity, PI 3-Kinase, p85, Pik3r1, Lipids, IR, insulin resistance, PI3K, phosphoinositide 3-kinase, SHORT syndrome, Short stature, Hyperextensibility of joints, Ocular depression, Rieger anomaly of the iris, and Teething delay, RER, Respiratory Exchange Ratio, HFD, High-Fat Diet

## Abstract

**Objective:**

Insulin signalling via phosphoinositide 3-kinase (PI3K) requires *PIK3R1-*encoded regulatory subunits. C-terminal *PIK3R1* mutations cause SHORT syndrome, as well as lipodystrophy and insulin resistance (IR), surprisingly without fatty liver or metabolic dyslipidaemia. We sought to investigate this discordance.

**Methods:**

The human pathogenic *Pik3r1* Y657^∗^ mutation was knocked into mice by homologous recombination. Growth, body composition, bioenergetic and metabolic profiles were investigated on chow and high-fat diet (HFD). We examined adipose and liver histology, and assessed liver responses to fasting and refeeding transcriptomically.

**Results:**

Like humans with SHORT syndrome, *Pik3r1^WT/Y657∗^* mice were small with severe IR, and adipose expansion on HFD was markedly reduced. Also as in humans, plasma lipid concentrations were low, and insulin-stimulated hepatic lipogenesis was not increased despite hyperinsulinemia. At odds with lipodystrophy, however, no adipocyte hypertrophy nor adipose inflammation was found. Liver lipogenic gene expression was not significantly altered, and unbiased transcriptomics showed only minor changes, including evidence of reduced endoplasmic reticulum stress in the fed state and diminished Rictor-dependent transcription on fasting. Increased energy expenditure, which was not explained by hyperglycaemia nor intestinal malabsorption, provided an alternative explanation for the uncoupling of IR from dyslipidaemia.

**Conclusions:**

*Pik3r1* dysfunction in mice phenocopies the IR and reduced adiposity without lipotoxicity of human SHORT syndrome. Decreased adiposity may not reflect *bona fide* lipodystrophy, but rather, increased energy expenditure, and we suggest that further study of brown adipose tissue in both humans and mice is warranted.

## Highlights

• SHORT syndrome features insulin resistance and reduced adiposity without dyslipidaemia and fatty liver.
• A mouse model with a pathogenic human PI 3-Kinase mutation recapitulates this uncoupling.
• Surprisingly, no adipose injury nor increased liver *de novo* lipogenesis is seen.
• Energy expenditure is increased, causing resistance to diet-induced obesity.
• This increases evidence for some beneficial metabolic effects of PI 3-Kinase inhibition.

## 1 Introduction

Adipose tissue is essential for metabolic health in the face of sustained positive energy balance. When adipose expansion is constrained, as in lipodystrophy, its capacity to sequester excess energy and serve as a physiological “energy buffer” is overwhelmed [1]. Injury and inflammation of any residual adipose tissue ensues, causing systemic inflammation and rerouting substrates to the liver, pancreas and muscle. Insulin resistance (IR), diabetes, aggressive fatty liver disease, and severely elevated plasma lipoprotein concentrations are thus common consequences of lipodystrophy [2].

SHORT syndrome (short stature, hyperextensibility of joints, ocular depression, Rieger anomaly of the iris, and teething delay) is a human monogenic syndrome including lipodystrophy [[3], [4], [5]]. This form of lipodystrophy is highly unusual - though associated with insulin resistant diabetes [[3], [4], [5]], this is uncoupled from fatty liver and dyslipidaemia [6]. SHORT syndrome also does not feature the suppressed plasma adiponectin concentration commonly seen in IR and other lipodystrophies [7], proving that some elements of the “insulin resistance syndrome” are dissociable.

SHORT syndrome is caused by mutations in *PIK3R1*; encoding components of the insulin signalling enzyme phosphoinositide 3-kinase (PI3K) [[3], [4], [5]]. The PI3K subtype required for insulin action comprises a p110α catalytic subunit bound to a regulatory subunit, three (p85α, p55α and p50α) of which are encoded by *PIK3R1*. *PIK3R1* products stabilise catalytic subunits, mediating their recruitment to receptor tyrosine kinases or their substrates, including the insulin receptor and IRS1 [8,9].

Before the discovery of *PIK3R1* mutations in SHORT syndrome, *Pik3r1* had been intensively studied in mice. Selective p85α deficiency [10,11] or deletion of p50α and p55α [12] surprisingly enhances insulin sensitivity, which has been accounted for by compensation by other regulatory subunits, and/or altered stoichiometry of catalytic and regulatory subunits. A critical insight from the study of SHORT syndrome was that the insulin signalling role of PIK3R1 could be severely attenuated by neomorphic mutations disrupting the C-terminal SH2 domain [[3], [4], [5]]. Such dominant negative PIK3R1 alleles, which severely reduce insulin-induced activation of PI3K without abolishing the expression of PI3K, offer the opportunity to interrogate, more precisely than has been possible to date, the *in vivo* pathophysiological roles of PI3K activity in IR.

In human lipodystrophy, *de novo* lipogenesis contributes significantly to liver triglyceride and plasma lipid content [13]. In contrast, mutations in the *INSR* gene encoding the insulin receptor produce extreme IR with normal plasma lipid profile, normal liver lipid content, and no increase in hepatic *de novo* lipogenesis [13]. Furthermore, murine models with knockout of any one of several components of the insulin signalling pathway (*Insr* [14], *Irs1* and *Irs2* [15], p85α and β [16], *Pik3ca* [17], or *Rictor* [18]) demonstrate that liver insulin signalling drives hepatic *de novo* lipogenesis. We thus hypothesised that in SHORT syndrome, the liver is protected from increased insulin-mediated lipogenesis, which is relatively unaffected by common IR [19], and that this explains the absence of dyslipidaemia and fatty liver despite severe IR and lipodystrophy.

We report a novel murine SHORT syndrome model caused by a human pathogenic allele, Y657∗, that reproduces uncoupling of metabolic dyslipidaemia from severe IR. Surprisingly, hepatic lipogenic transcriptional programmes were minimally perturbed, although metabolic inflexibility with a dyscoordinated fasting response was seen. Adipose tissue accumulation was reduced on high-fat feeding. We suggest that this is related to increased energy expenditure, likely via brown adipose tissue, rather than lipodystrophy, with hypolipidemia attributable to increased fuel oxidation. This potential explanation for the unusual IR subphenotype of SHORT syndrome warrants testing in humans with SHORT syndrome or other proximal insulin signalling defects.

## 2 Materials and methods

### 2.1 Mice generation and maintenance

The *Pik3r1* Y657∗ mutation and a neomycin resistance cassette flanked by LoxP sites were introduced into C57Bl/6 embryonic stem cells by homologous recombination before injection into Bl/6J blastocysts (Supplemental Figure S1). Animals were kept on a C57Bl/6J background, backcrossed at least three times, and housed on a 12-h-light/12-h-dark cycle at 23 °C, with *ad libitum* food and water access. Feeding was with chow (Catalogue no. 105, Safe diets) or 45% fat diet (45% fat, 35% Carbohydrate and 25% protein) (Catalogue no. D12451, Research diet, Inc). Experiments were carried out under the UK Home Office Animals (Scientific Procedures) Act 1986, following University of Cambridge ethical review.

### 2.2 Assessment of growth, body composition, and energy homeostasis

Body composition was determined by time-domain NMR with Bruker's minispec analyser. For food intake and energy expenditure measurement, male mice were acclimatized for 1 week to single housing then given chow or 45% fat diet for 10 days. Food intake was measured daily and energy expenditure measured by indirect calorimetry [20]. Locomotor activity was quantified as beam breaks over 48 h. Faecal energy content was measured by combusting dried faeces from 48 h calorimeter runs in an IKA Calorimeters Oxygen Bomb calorimeter (IKA C1).

### 2.3 *In vivo* metabolic studies

In fasting/refeeding studies, mice were fed chow *ad libitum* until 16 weeks of age before 16 h fasting, then 6 h refeeding with chow. Tissues were harvested and snap frozen prior to overnight fasting, or after fasting or after refeeding. For the study of insulin action, 16 h-fasted mice (16 weeks old) were injected with 2 U/kg insulin intraperitoneally and tissues collected after 10 min and snap frozen. Glucose tolerance testing was performed on overnight (16 h) fasted mice using 2 g/kg 20% glucose via oral gavage. Insulin tolerance testing was done by giving 0.5 U/kg intraperitoneal insulin to 6 h-fasted mice. For lipid tolerance testing, 16 h-fasted mice were administered 10 mL/kg olive oil by gavage.

Hyperinsulinemic euglycemic clamp studies and subsequent sample processing and analysis were conducted as described previously [21] on 16 week old mice after overnight fasting. The clamps used a priming dose of human insulin (0.7 mU), followed by constant insulin and [3-^3^H]-d-glucose infusion at 7 mU/h and 0.72 μCi/h, respectively.

### 2.4 Study of tissue insulin responsiveness *ex vivo*

Measurement of insulin action on isolated Soleus and Extensor Digitorum Longus was undertaken in 16-week-old male mice as described [22]. For primary preadipocyte differentiation studies, fat pads from 8 week-old male mice were isolated and cultured as described [20], but without 5-Triiodo-l-thyronine.

### 2.5 Biochemical assays

Blood was collected by cardiac puncture and plasma snap frozen before assays detailed in Supplemental Table 3. Glycogen hydrolysis in 1 M HCl (2.5 h at 100 °C), NaOH neutralization and fluorometric glucose analysis were used to determine the glycogen content of snap-frozen liver [23]. Hepatic triglyceride determination was used in the Triglyceride Colorimetric Assay kit (Cayman). Hepatic cholesterol was extracted from 10 mg of liver using a 400 μL chloroform:isopropanol:NP-40 mixture (7:11:0.1) before centrifugation, 30 min vacuum drying (50 °C) and dissolving in phosphate-buffered saline (PBS). The samples were assayed by Siemens Healthcare Diagnostics kit.

### 2.6 Metabolomic studies

Liver metabolites were extracted from tissue snap-frozen after dissection using a reported methanol/chloroform method [24]. Aqueous metabolites were further extracted as described in [24] and analysed using a Vanquish ultra-high performance liquid chromatography (UHPLC) system and TSQ Quantiva triple quadrupole mass spectrometer (Thermo Scientific) with compounds directly infused. Parameter optimisation used 1 μM standard solutions in a chromatographic buffer. Optimal mass spectrometry parameters and mass transitions were generated by automatic MassLynx™ (Version 1.4, Waters) protocols or, if standards were unavailable, deduced from known analogue parameters.

### 2.7 Assessment of *de novo* lipogenesis

Sixteen week chow-fed mice were fasted overnight, injected intraperitoneally with deuterated saline (24 μL/g), and given high carbohydrate diet (Harlan Teklad Diets, Cat no. TD88232) and 4% deuterated water (Goss Scientific Instruments Ltd, Cat no. DLM-2259) from 1 to 24 h later. Subsequent to culling, livers were snap-frozen and 50 mg homogenised in 600 μL methanol–chloroform (2:1) before extraction and analysis as described [25].

### 2.8 Histology

The sizes of at least 1000 adipocytes per genotype from 16-week-old chow-fed mice were measured in Formalin-fixed, paraffin-embedded (FFPE) tissue as described [26]. Adipose Treg cells were isolated and counted by FACS, also as reported [27].

### 2.9 Gene expression analysis

For protein studies fresh tissues were snap-frozen in liquid nitrogen, and homogenised using MD ceramic beads in RIPA buffer with proteinase/phosphatase inhibitors (liver, muscle) (Roche) or mortar and pestle in liquid nitrogen before dissolving in buffer (adipose). Protein concentrations were determined by BCA assay (BioRad). Western blotting employed the Novex gel system (Thermo Fisher Scientific). Antibodies are shown in Supplemental Table S4. Quantification employed the BioRad image system.

Total RNA was extracted and reverse transcribed for qPCR as previously described [28]. Target expression was normalised to the geometric mean of 4 housekeeping genes (Ywhaz, Ppia, B2m and Eef1a1) (Supplemental Table S4 for primer/probe sequences). RNA sequencing protocols and analysis have previously been reported [29].

### 2.10 Statistical analysis

D'Agostino & Pearson or Shapiro–Wilk tests were performed to test for Gaussian distribution of data. For Gaussian data, unpaired two-tailed Student's t tests for two group comparison and ANOVA with *post hoc* for multiple group comparison were performed using GraphPad Prism (GraphPad Software). For non-Gaussian data Mann–Whitney testing was used for two group comparison and Kruskal–Wallis and Dunn's multiple comparison testing were used for multiple group analysis using GraphPad Prism (GraphPad Software). Tissue weights, food consumption and energy expenditure were analysed by ANCOVA using XLSTAT (Addinsoft).

### 2.11 Data and resource availability

All transcriptomic data were deposited in GEO under accession number GSE153431. Other datasets, and the *Pik3r1*^*WT/Y657*∗^ mice generated during the current study are available from the corresponding author upon reasonable request.

## 3 Results

### 3.1 Growth and development of Pik3r1 Y657∗ knock-in mice

Mice harbouring the *Pik3r1* Y657∗ mutation were generated by homologous recombination-based gene targeting (Figure S1A). Immunoblotting confirmed the expression of truncated p85α at greater amounts than full length protein in most tissues, likely due to the loss of an ubiquitylation motif located beyond the nonsense mutation in the C-terminal [30]. No compensatory change in PI3K p85β subunit was seen. p110α subunit expression was reduced in subcutaneous white adipose tissue, and p110β expression was reduced in liver, subcutaneous and epididymal white adipose tissue (Figure S1B).

**Figure 1:**
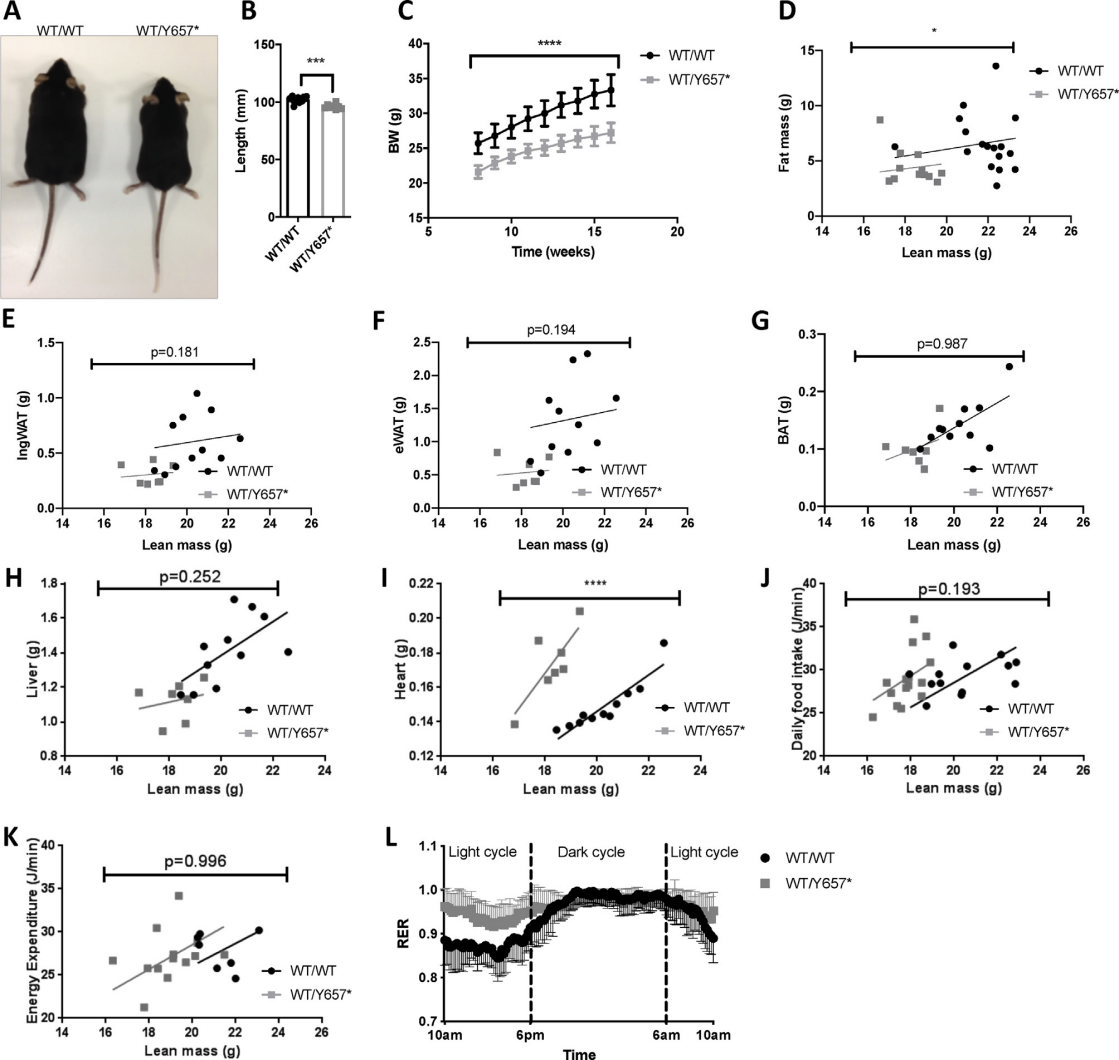
**Effect of *Pik3r1* Y657∗ on prenatal development and postnatal growth.** (A) Representative image of *Pik3r1*^*WT/WT*^ (WT/WT) and *Pik3r1*^*WT/Y657*∗^ (WT/Y657∗) mice at 18 weeks old. (B) Body lengths (nose to anus) at 18 weeks of *Pik3r1*^*WT/WT*^ and *Pik3r1*^*WT/Y657*∗^ mice (*n* = 11 and 18 respectively). (C) Bodyweight increase from 8 to 16 weeks of *Pik3r1*^*WT/WT*^ and *Pik3r1*^*WT/Y657*∗^ (*n* = 16 and 12 respectively). (D) The relationship between lean and fat mass of *Pik3r1*^*WT/WT*^ and *Pik3r1*^*WT/Y657*∗^ mice (*n* = 16 and 12 respectively). Masses of (E) inguinal adipose tissue (IngWAT) (F) Epididymal adipose tissue (eWAT), (G) Brown adipose tissue (BAT), (H) Liver, and (I) Heart of *Pik3r1*^*WT/WT*^ and *Pik3r1*^*WT/Y657*∗^ mice (*n* = 11 and 7 respectively). (J) Food intake (*n* = 13 for *Pik3r1*^*WT/WT*^ and *n* = 14 for *Pik3r1*^*WT/Y657*∗^), (K) Energy expenditure and (L) Respiratory exchange ratio (RER) (*n* = 7 for *Pik3r1*^*WT/WT*^ and *n* = 13 for *Pik3r1*^*WT/Y657*∗^) of wild-type and heterozygous mice assessed at 18 weeks old. All data shown are from male mice. Masses and energy expenditure are shown relative to total lean mass, and were analysed statistically by ANCOVA. ∗ = *p* < 0.05; ∗∗∗ = *p* < 0.001; ∗∗∗∗ = *p* < 0.0001. Mean ± SD are shown for plots in (B), (C) and (L).

No *Pik3r1*^*Y657*∗/*Y657*∗^ embryos were identified beyond E11.5. At E11.5 they were smaller, with poorly developed limb buds and reduced eye pigmentation. Heterozygous embryos were smaller from E15.5 (Figure S2A–E). *Pik3r1*^*WT/Y657*∗^ mice were born at the expected frequency, but showed impaired linear growth and body weight on chow (Figure 1A–C). At 16 weeks, there was a small decrease in whole body adiposity (Figure 1D), however, no difference was found in epididymal or inguinal white adipose nor interscapular brown adipose depot weights when lean mass was taken into account (Figure 1E–G). Plasma leptin concentrations were similar in male *Pik3r1*^*WT/Y657*∗^ and wild-type mice (3.3 ± 1.2 *vs* 2.6 ± 0.3 μg/L on fasting (*n* = 9,10; NS); 10.3 ± 2.2 *vs* 15.9 ± 3.9 μg/L on *ad libitum* feeding (*n* = 13,13; NS)). Liver weights were indistinguishable (Figure 1H), but hearts were heavier in *Pik3r1*^*WT/Y657*∗^ mice (Figure 1I). No difference in food consumption nor energy expenditure was seen at 16 weeks on chow (Figure 1J,K), however, the respiratory exchange ratio (RER) was higher during the light phase in *Pik3r1*^*WT/Y657*∗^ mice, blunting the circadian fluctuation (Figure 1L). Similar differences were seen between heterozygous and wild-type females for all variables assessed in both sexes.

### 3.2 Response of *Pik3r1*^*WT/Y657*∗^ mice to a high-fat diet

**Figure 2:**
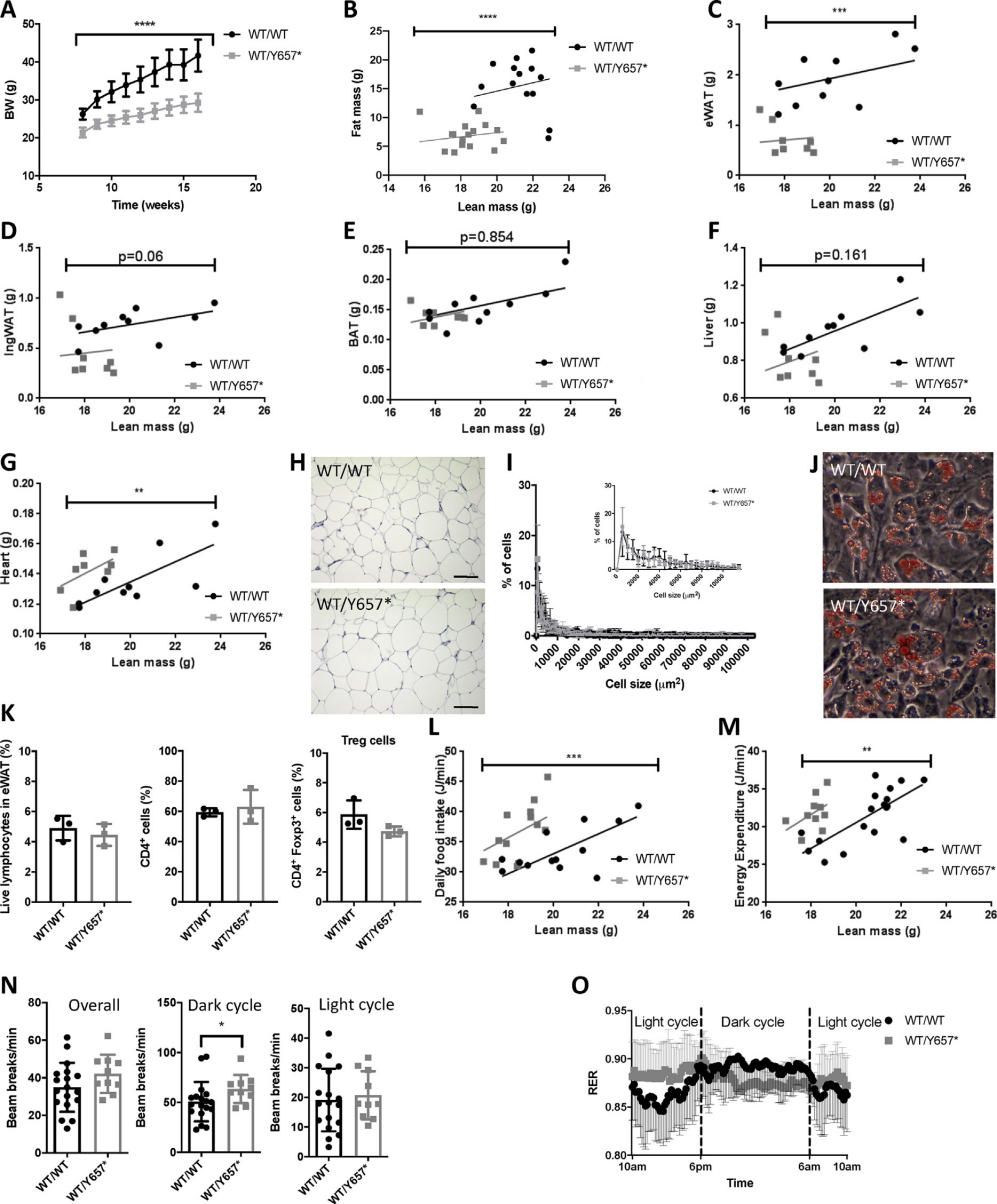
**Response of *Pik3r1*^*WT/Y657*∗^ mice to a palatable 45% fat diet.** (A) Bodyweight increase from 8 to 16 weeks of *Pik3r1*^*WT/WT*^ (WT/WT) and *Pik3r1*^*WT/Y657*∗^ (WT/Y657∗) (*n* = 16 and 12 respectively). (B) The relationship between lean and fat mass of *Pik3r1*^*WT/WT*^ and *Pik3r1*^*WT/Y657*∗^ mice (*n* = 16 and 12 respectively). Masses of (C) Epididymal adipose tissue (eWAT), (D) Inguinal adipose tissue (IngWAT), (E) Brown adipose tissue (BAT), (F) Liver, and (G) Heart of *Pik3r1*^*WT/WT*^ and *Pik3r1*^*WT/Y657*∗^ mice (*n* = 12 for both genotypes). (H) Representative histological appearance of haematoxylin and eosin-stained eWAT from *Pik3r1*^*WT/WT*^ and *Pik3r1*^*WT/Y657*∗^ mice. Scale bars = 100 μm (I) Adipocyte size distribution in eWAT based on quantification of >1000 cells per genotype from 4 wild-type and 4 heterozygous mice. The inset shows a zoomed-in view of the percentage of cells smaller than 10 000 μm^2^. (J) Representative images of *ex vivo* differentiated stromal vascular cells from ingWAT stained with Oil Red O. (K) Percentage of CD4^+^ and regulatory T cells in the eWAT (*n* = 3 for *Pik3r1*^*WT/WT*^ and *n* = 3 for *Pik3r1*^*WT/Y657*∗^). (L) Food intake (*n* = 13 for *Pik3r1*^*WT/WT*^ and *n* = 14 for *Pik3r1*^*WT/Y657*∗^) and (M) Energy expenditure (*n* = 17 for *Pik3r1*^*WT/WT*^ and *n* = 10 for *Pik3r1*^*WT/Y657*∗^) of wild-type and heterozygous mice assessed at 18 weeks old. All masses and energy expenditure are shown relative to total lean mass, and were analysed statistically by ANCOVA. (N) Locomotor activity of *Pik3r1*^*WT/WT*^ and *Pik3r1*^*WT/Y657*∗^ mice (*n* = 17 and *n* = 10 respectively). (O) Respiratory exchange ratio (RER) (*n* = 17 for *Pik3r1*^*WT/WT*^ and *n* = 10 for *Pik3r1*^*WT/Y657*∗^) of wild-type and heterozygous mice assessed at 18 weeks old. All data shown are from male mice. Mean ± SD are shown. ∗*p* < 0.05, ∗∗*p* < 0.01, ∗∗∗*p* < 0.001 and ∗∗∗∗*p* < 0.0001.

To assess whether placing a greater load on adipose tissue in *Pik3r1*^*WT*/*Y657*∗^ mice would unmask lipodystrophy, mice were fed a 45% fat diet (HFD) from 8 weeks old. ${\text{Pik3r1}}^{{WT/Y657}^{*}}$ mice showed reduced weight gain and body fat over the 8 weeks of study (Figure 2A,B). Epididymal white adipose tissue was most severely affected (Figure 2C), with no significant difference in inguinal nor brown adipose tissue (Figure 2D,E). Lean mass did not increase at a greater rate in *Pik3r1*^*WT/Y657*∗^ mice (Figure S3A,B), and liver weights also did not differ, while heart weights were elevated in heterozygotes, as on chow (Figure 2F,G). Consistent with reduced adiposity, *Pik3r1*^*WT/Y657*∗^ mice had lower serum leptin concentrations when fed (37.7 ± 7.3 *vs* 11.3 ± 2.0 μg/L (*n* = 15,11; *p* = 3 × 10^−3^)), but not when fasted (15.9 ± 3.9 *vs* 10.3 ± 2.2 μg/L (*n* = 13,13; not significant)), while adiponectin concentrations were unchanged (Table 1).

**Table 1:** Fasting plasma biochemical profile of male *Pik3r1*^*WT/WT*^ and *Pik3r1*^*WT/Y657*∗^ mice. See also Supplemental Table S1 for fasting biochemical profile for chow-fed female mice. N.D. = not determined. Statistical comparisons were undertaken using Student's *t*-test.

	Chow						45% Fat diet					
	Fasting			Fed			Fasting			Fed		
	*Pik3r1*^*WT/WT*^ (*n* = 10)	*Pik3r1*^*WT/Y657*∗^ (*n* = 9)	*p*	*Pik3r1*^*WT/WT*^ (*n* = 10)	*Pik3r1*^*WT/Y657*∗^ (*n* = 14)	*p*	*Pik3r1*^*WT/WT*^ (*n* = 13)	*Pik3r1*^*WT/Y657*∗^ (*n* = 13)	*p*	Pik3r1^WT/WT^ (*n* = 15)	*Pik3r1*^*WT/Y657*∗^ (*n* = 11)	*p*
Glucose (mmol/L)	10.2 ± 1.1	9.0 ± 1.1	0.43	12.5 ± 0.7	13.2 ± 0.5	0.48	9.7 ± 0.8	7.9 ± 0.5	0.06	*12.6 ± 0.5*	*9.4 ± 0.5*	*4 × 10*^*−4*^
Insulin (pmol/L)	*138 ± 21*	*603 ± 202*	*0.05*	*229 ± 33*	*3393 ± 578*	*1.9 × 10*^*−4*^	469 ± 77	693 ± 102	0.09	424 ± 73	1313 ± 494	0.104
Adiponectin (mg/L)	*30.5 ± 1.1*	*26.7 ± 1.3*	*0.03*	33.2 ± 1.5	35.6 ± 1.0	0.2	37.0 ± 1.1	40.6 ± 1.5	0.07	40.3 ± 2.2	42.8 ± 1.1	0.3
Total cholesterol (mmol/L)	*2.7 ± 0.1*	*2.3 ± 0.1*	*0.01*	*2.6 ± 0.1*	*2.0 ± 0.1*	*6 × 10*^*−3*^	*4.2 ± 0.2*	*3.0 ± 0.3*	*0.01*	*4.7 ± 0.3*	*1.7 ± 0.1*	*1.0 × 10*^*−7*^
HDL cholesterol (mmol/L)	*1.5 ± 0.1*	*1.1 ± 0.1*	*0.02*	*1.6 ± 0.1*	*1.3 ± 0.1*	*0.04*	*1.8 ± 0.2*	*1.2 ± 0.2*	*0.02*	*2.3 ± 0.1*	*0.9 ± 0.1*	*2.7 × 10*^*−8*^
Triglycerides (mmol/L)	*1.5 ± 0.1*	*1.0 ± 0.1*	*0.01*	*1.6 ± 0.1*	*1.1 ± 0.1*	*0.01*	1.5 ± 0.1	1.4 ± 0.1	0.15	*1.0 ± 0.1*	*0.6 ± 0.02*	*3.5 × 10*^*−6*^
NEFA (mmol/L)	*2.0 ± 0.2*	*1.2 ± 0.1*	*5 × 10*^*−3*^	1.5 ± 0.1	1.3 ± 0.2	0.3	*2.5 ± 0.2*	*1.9 ± 0.1*	*0.05*	*1.5 ± 0.1*	*0.8 ± 0.1*	*7 × 10*^*−5*^
VLDL (mg/mL)	N.D.	N.D.	N.D.	7.9 ± 0.3	7.4 ± 1.2	0.25	N.D.	N.D.	N.D.	10.5 ± 0.5	11.9 ± 0.8	0.13

Given reduced adipose expansion, we subsequently sought histological evidence of “adipose failure”, namely adipocyte hypertrophy and inflammation, which would be expected in lipodystrophy when adipose expansion is pathologically constrained, causing adipose injury on positive energy balance. The histological appearance and adipocyte size distribution in epididymal adipose tissue was indistinguishable between genotypes (Figure 2H,I), however with no evidence of inflammation, nor fibrosis. Consistent with the lack of histological evidence of adipose overload, *ex vivo* differentiation of preadipocytes from inguinal or epididymal depots was unaltered (Figure 2J). Finally, given the role of *Pik3r1* in lymphocyte function, and the important role of adipose-resident T regulatory (T_reg_) cells in energy homeostasis, the adipose content of T_reg_ cells was assessed by FACS. No major difference was observed between genotypes (Figure 2K).

Given these findings, which are at odds with a true lipodystrophy, we then assessed whether reduced adipose accretion rather reflected altered energy homeostasis. *Pik3r1*^*WT/Y657*∗^ mice exhibited increased food intake (Figure 2L) and energy expenditure (Figure 2M) when the size of the animals was taken into account, but locomotor activity was unchanged across 24 h (Figure 2N). Markedly reduced accumulation of adipose tissue despite increased food intake, and without sustained hyperglycaemia, demonstrates severely reduced food efficiency. This indicates that increased food intake is a compensatory response to chronically increased energy expenditure rather than a primary phenomenon. As on chow, the circadian RER profile was blunted, with higher values in the dark phase and no discernible difference between light and dark phases for *Pik3r1*^*WT/Y657*∗^ mice (Figure 2O), suggesting greater carbohydrate oxidation in the dark phase.

### 3.3 *Pik3r1*^*WT/Y657*∗^ mice are severely insulin resistant

At 12 weeks old, neither male nor female *Pik3r1*^*WT/Y657*∗^ mice on chow were hyperglycaemic compared to controls, but plasma insulin concentrations were raised, more severely on feeding (Tables 1 and S1). On high-fat feeding, male *Pik3r1*^*WT/Y657*∗^ mice remained hyperinsulinemia, but the difference between mutant and control mice was abolished by the greater increase in insulin concentrations in wild-type mice. Blood glucose concentration was paradoxically lower in fed *Pik3r1*^*WT/Y657*∗^ mice on HFD. Plasma adiponectin concentrations in fasted chow-fed *Pik3r1*^*WT/Y657*∗^ mice were lower than in wild-type controls, however no significant difference was seen in other conditions, despite the severe IR of heterozygous mice (Table 1). In *Pik3r1*^*WT/Y657*∗^ mice on HFD, adiponectin normalised to body fat was higher than in controls, reminiscent of the preserved adiponectin seen in severely insulin-resistant patients with SHORT syndrome [7]. Normalised plasma leptin concentrations, in contrast, were lower (Figure S3C,D).

**Figure 3:**
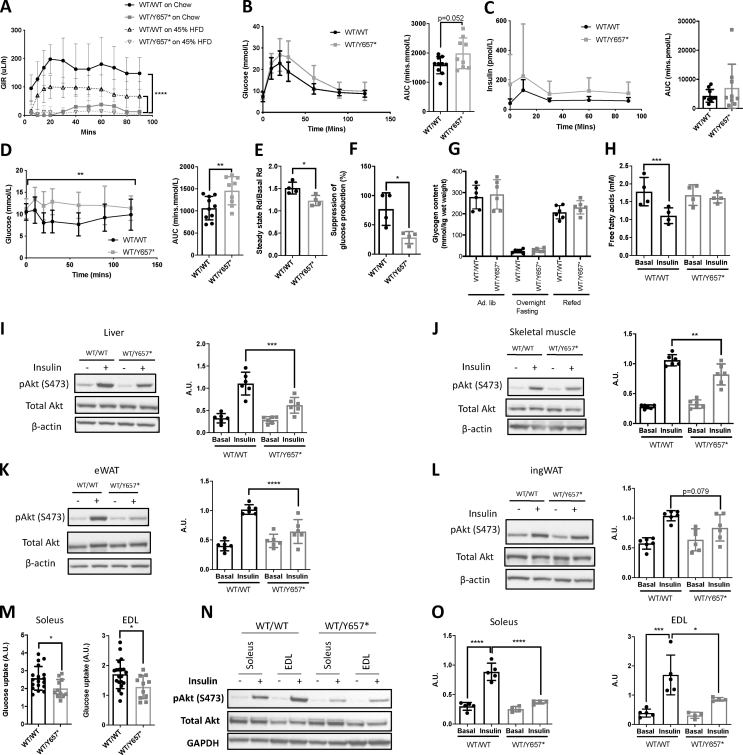
***Pik3r1*^*WT/Y657*∗^ mice show systemic and tissue-level insulin resistance.** (A) Glucose infusion rates during hyperinsulinemic euglycemic clamping of *Pik3r1*^*WT/WT*^ (WT/WT) and *Pik3r1*^*WT/Y657*∗^ (WT/Y657∗) mice on chow (*n* = 4 and 4) or 45% fat diet (*n* = 10 and 11) at 16 weeks old. (B) Oral glucose tolerance test (OGTT) and corresponding comparison of areas under the curves (AUC) of *Pik3r1*^*WT/WT*^ and *Pik3r1*^*WT/Y657*∗^ mice on chow at 12 weeks old (*n* = 10 and 9). (C) Insulin concentrations and AUC for the same OGTT as in (B). (D) Insulin tolerance test and AUC comparison for the same mice 1 week later. (E) Glucose disposal and (F) suppression of hepatic glucose output by insulin during hyperinsulinemic euglycemic clamping of *Pik3r1*^*WT/WT*^ and *Pik3r1*^*WT/Y657*∗^ mice on chow at 18 weeks old (both *n* = 4). (G) Glycogen content of livers during a fasting–refeeding cycle in chow fed animals at 16 weeks old (both *n* = 6). (H) Plasma non-esterified free fatty acid concentrations during hyperinsulinemic euglycemic clamping (both genotypes *n* = 4). (I)–(L) Representative images of immunoblots and corresponding quantifications of tissue lysates from mice injected intraperitoneally with 2 U/kg insulin 10 min prior to sacrifice, showing pAkt^Ser473^, total Akt and their ratio: (I) Liver, (J) Skeletal muscle (K) eWAT, (L) ingWAT (*n* = 6 per genotype and condition). (M) Insulin-induced fold increase of glucose uptake into *ex vivo* incubated soleus (*n* = 18 for *Pik3r1*^*WT/WT*^, *n* = 11 for *Pik3r1*^*WT/Y657*∗^) and Extensor Digitorum Longus (EDL) (*n* = 20 and 11). (N) Representative immunoblots of Soleus and EDL lysates from the same paradigm as in (M). (O) Quantification of pAkt^Ser473^ to total Akt ratios from soleus and EDL immunoblots (*n* = 5 and 4 for both). Data are from male mice. Quantitative data are presented as mean ± SD. ∗ = *p* < 0.05, ∗∗ = *p* < 0.01, ∗∗∗ = *p* < 0.001 and ∗∗∗∗ = *p* < 0.0001.

Hyperinsulinemic euglycemic clamps were undertaken on chow- and HFD-fed male mice. Chow-fed *Pik3r1*^*WT/Y657*∗^ mice required a steady state glucose infusion rate of only one eighth that required by wild-type mice, confirming severe IR (Figure 3A). On HFD, *Pik3r1*^*WT/Y657*∗^ mice remained extremely insulin resistant, however the difference between genotypes was much smaller than on chow due to increased IR in wild-type animals (Figure 3A). Further studies were thus carried out on chow. Glucose and insulin excursions on oral glucose tolerance testing showed a trend towards an increase in male *Pik3r1*^*WT/Y657*∗^ mice (Figure 3B,C), but were increased in female mice (Figure S4A,B), while the hypoglycaemic response to insulin was attenuated in both sexes (Figures 3D and S4C). Clamp studies in males using isotopic tracers showed glucose disposal to be 19% lower in *Pik3r1*^*WT/Y657*∗^ mice (Figure 3E) while suppression of hepatic glucose production by hyperinsulinemia was 49% lower compared to wild type littermates (Figure 3F). Liver glycogen content was similar between genotypes (Figure 3G). Insulin infusion lowered plasma free fatty acid concentrations in wild-type mice, but little suppression was seen in heterozygous mice, consistent with impaired suppression of adipose lipolysis (Figure 3H).

Intraperitoneal insulin in male mice strongly induced Akt phosphorylation in the liver, skeletal muscle and epididymal and inguinal adipose tissue of wild-type controls, as expected, and this was reduced in *Pik3r1*^*WT/Y657*∗^ mice (Figures 3I–L and S5A–D). As muscle insulin sensitivity was previously reported not to be reduced in *Pik3r1*^*WT/R649W*^ mice [31], insulin responsiveness of soleus and extensor digitorum longus (EDL) muscles was assessed *ex vivo*. Soleus and EDL muscle from *Pik3r1*^*WT/Y657*∗^ mice both showed a 1.3-fold reduction in insulin-stimulated deoxyglucose uptake compared to wild-type muscle (Figure 3M). Insulin-induced Akt phosphorylation was also markedly reduced in both types of muscle *ex vivo* to a much more significant degree than *in vivo* (Figures 3N,O and S5E). This finding of muscle insulin resistance using several techniques, although contrasting with clamp studies reported previously, is consistent with the robust expression of *Pik3r1* gene products in skeletal muscle.

### 3.4 *Pik3r1*^*WT/Y657*∗^ mice are hypolipidemic

Having demonstrated that, as in SHORT syndrome, heterozygous *Pik3r1*^*WT/Y657*∗^ mice show severe IR and reduced adiposity, we assessed whether, like humans, they do not exhibit fatty liver nor metabolic dyslipidaemia. In fed and fasting states, on either chow or HFDs, *Pik3r1*^*WT/Y657*^^∗^ mice showed hypolipidemia, with lower plasma total cholesterol and HDL cholesterol (Table 1). This was most striking in fed mice on HFD, mirroring the lower glucose seen in *Pik3r1*^*WT/Y657*∗^ mice in this state. Plasma triglyceride concentrations were also lower in heterozygous animals in all states except when the fasting state after HFD. Plasma free fatty acid concentrations were lower in all states except in fed animals maintained on chow. Similar hypolipidemia was seen in chow-fed female mice on fasting (Table S1). No difference was seen in VLDL concentrations in chow- or high fat-fed mice (Table 1).

**Figure 4:**
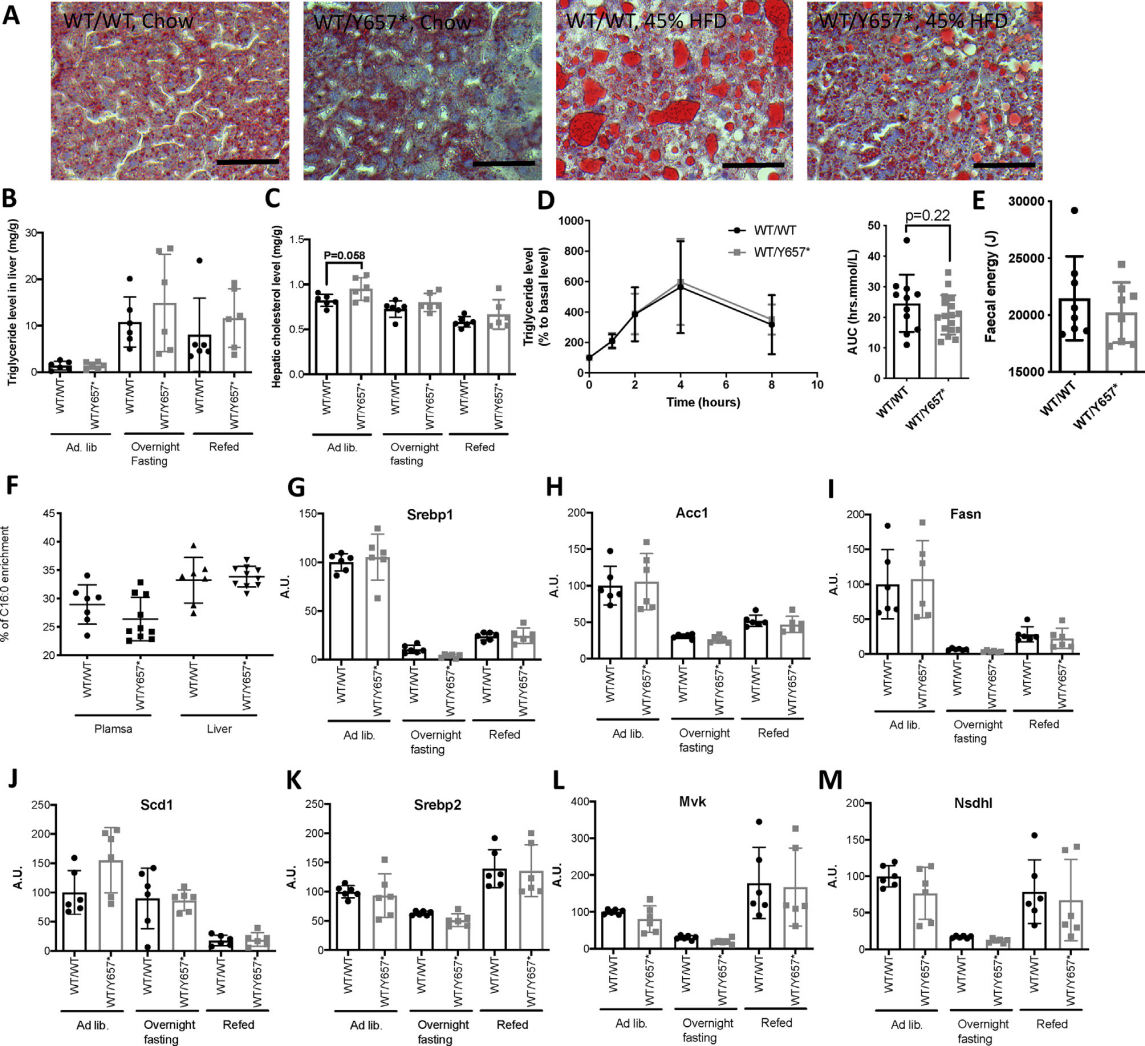
**Lipid handling and liver phenotype of *Pik3r1*^*WT/Y657*∗^ mice.** (A) Representative images of Oil-Red-O-stained livers of chow-fed and 45% fat diet-fed *Pik3r1*^*WT/WT*^ (WT/WT) and *Pik3r1*^*WT/Y657*∗^ (WT/Y657∗) mice. Scale bars = 200 μm. (B) Hepatic triglyceride and (C) Hepatic total cholesterol concentration during a fasting refeeding cycle of chow fed mice at 16 weeks old (*n* = 6 per genotype). (D) Lipid tolerance testing and comparison of areas under the curve (AUC), also of chow-fed mice, at 16 weeks old. Triglyceride concentrations were equalised at baseline by matching the difference between genotypes with the lower values of heterozygous mice, and the same fixed correction was applied to all points on the graph (*n* = 11 and *n* = 17 for wild-type and heterozygous mice respectively). (E) Faecal energy content determined by bomb calorimetry of chow fed mice at 16 weeks old (*n* = 8 and 8). (F) De novo palmitate measured by stable deuterium enrichment (*n* = 7 and *n* = 10 for wild-type and heterozygous mice respectively). (G)–(M) Liver mRNA expression, determined by quantitative real time PCR of (G) *Srebp1* and its transcriptional targets (H) *Acc1*, (I) *Fasn*, and (J) *Scd1*, and of (K) *Srebp2* and its transcriptional targets (L) *Mvk* and (M) *Nsdhl* in chow fed mice during a fasting refeeding cycle at 16 weeks old (*n* = 6 per genotype per condition). All data shown are from male mice. Numerical data are presented as mean ± SD.

Despite severe IR in *Pik3r1*^*WT/Y657*∗^ mice, no difference was seen in liver triglyceride content compared to controls either on chow or HFD, assessed by Oil-Red-O staining and biochemical quantification (Figure 4A,B). Liver cholesterol content was also the same in chow-fed animals of both genotypes (Figure 4C). Oral lipid tolerance testing revealed similar triglyceride excursion after lipid loading in both genotypes (Figure 4D), and bomb calorimetry of faeces on chow showed the same energy content (Figure 4E), excluding abnormal intestinal lipid handling as the explanation for hypolipidemia and reduced adiposity.

Insulin stimulates hepatic *de novo* lipogenesis and cholesterogenesis via *Srebp1* and *Srebp2* respectively. In prevalent IR, hepatic *de novo* lipogenesis is enhanced, and the mismatch between this and the reduced hypoglycaemic action of insulin is evidence for “partial IR” in metabolic dyslipidaemia [19]. Liver PI3K mediates the action of insulin on lipogenesis, with hypolipidemia despite IR seen in numerous models in which hepatic insulin/Irs/PI3K/Akt2/mTORC2 signalling is impaired [[14], [15], [16],^18^,^32^]. Based on these findings, we hypothesised that the hypolipidemia seen in *Pik3r1*^*WT/Y657*∗^ mice would be accounted for by reduced hepatic *de novo* lipogenesis, as in SHORT syndrome due to PIK3R1 Y657∗ [7]. In keeping with this, no increased *de novo* synthesis of palmitate could be measured in heterozygotes despite fasting hyperinsulinemia and refeeding with a high carbohydrate diet (Figure 4F). Liver mRNA expression of *Srebp1c*, *Srebp2* and key target genes (*Acc1*, *Fasn*, *Scd1 and Mvk*, *Nsdhl, respectively*) was also determined in chow-fed animals in fed, fasting and refed states, and no difference was seen in the expression of any of these genes under any nutritional conditions (Figure 4G–M).

Other transcriptional regulators relevant to hypolipidemia include carbohydrate response element binding protein (*Chrebp*) and *Xbp1*, with the latter binding p85α and facilitating its nuclear translocation [31,33,34]. A sentinel *Chrebp*-responsive gene (*Pklr*) showed mild but significant reduction of mRNA in the fasting state, but mRNA of two *Xbp1* target genes (*Acacb* and *Dgat2*) showed no reduction during a fasting refeeding cycle (Figure S6A–C). It has also been proposed that modulation of the ratio between Glucose-6-phosphatase (*G6pc*) and Glucokinase (*Gck*) toggles hepatocyte glucose flux between lipogenesis and glucose production, and that this operates before transcriptional changes in lipogenic transcription factors [35]. However, although an increase in *G6pc*:*Gck* liver transcript levels was seen on fasting of wild-type mice, the ratio was suppressed in *Pik3r1*^*WT/Y657*∗^ mice, as reported in mice on Western-type diet [35] (Figure S6D–F). We conclude that the consistent hypolipidemia seen in *Pik3r1*^*WT/Y657*∗^ mice does not correlate with reduced insulin-induced lipogenesis in the liver, suggesting that the reduced blood lipid concentrations are not explained by attenuation of insulin's lipogenic actions.

**Figure 5:**
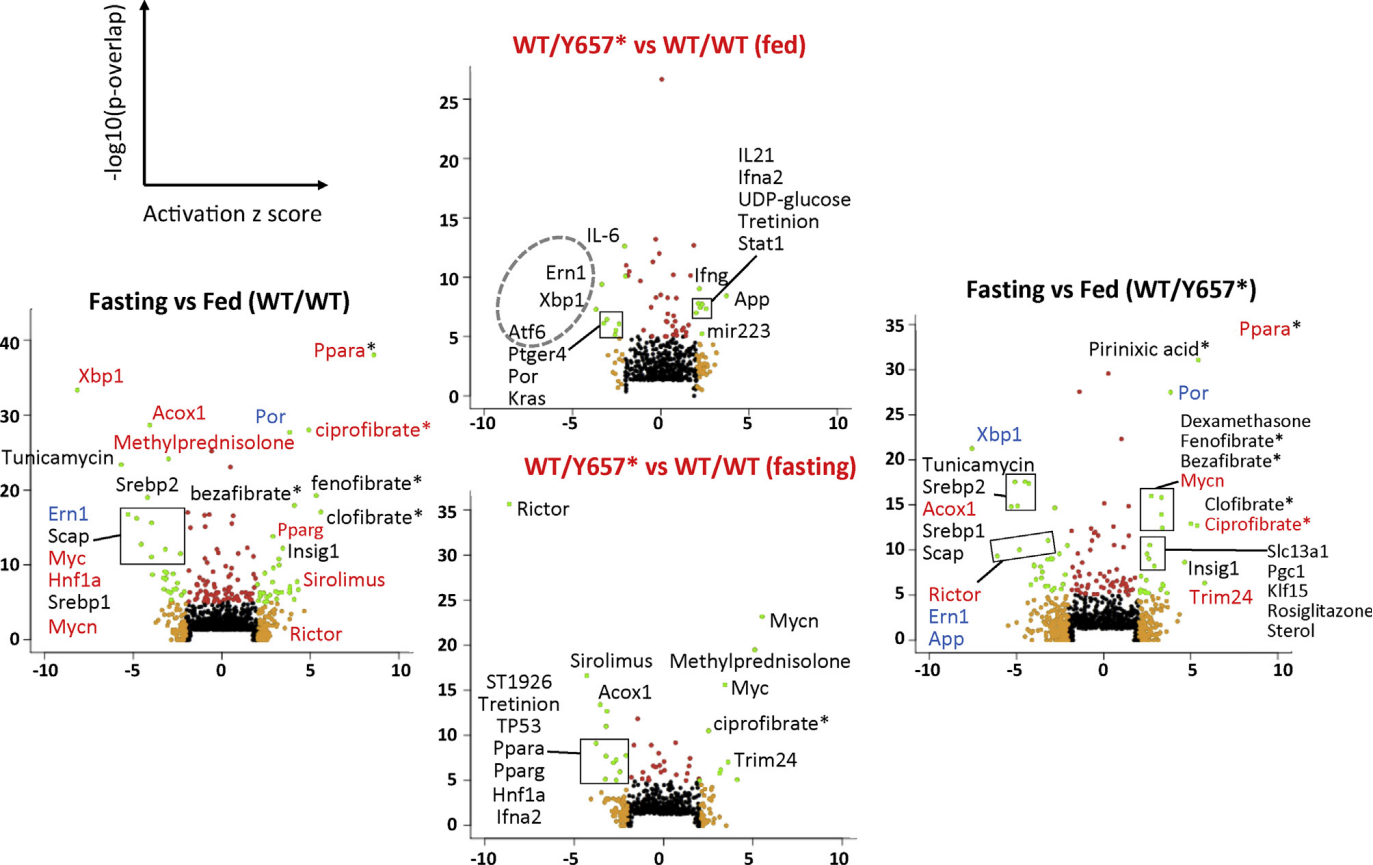
**Pathway analysis of liver transcriptomes of fed and fasted mice.** Volcano plots are shown for predicted upstream regulators derived from Ingenuity® Pathway Analysis (IPA®) of transcriptomes of fed and fasted male *Pik3r1*^*WT/Y657*∗^ (*n* = 6,6) mice and wild-type littermates (*n* = 6,6). Outside plots show regulators showing differential activity in the fasting state compared to the fed state for wild-type (WT; left) and *Pik3r1*^*WT/Y657*∗^ (right) mice. Central plots show regulators with differential activity in *Pik3r1*^*WT/Y657*∗^ vs WT mice in the fed state (top) and fasting state (bottom). The green dots represent data point with an activation *z* score <−2 or >+2 and a *p*-value <1 × 10^−5^. Regulators showing differential activity in either genotype-based comparison are coloured red (activation *z* score > +2 in *Pik3r1*^*WT/Y657*∗^ vs WT mice) or blue (activation *z* score < −2 in *Pik3r1*^*WT/Y657*∗^ vs WT mice) in the outside plots showing differences based on nutritional state. Statistical analysis was performed using a general linear model with Bonferroni correction.

Given the lack of transcriptional perturbation of known lipogenic factors, global liver transcriptomic analysis was performed in fed and 16 h fasted states. Genotypes were compared in each state, and fasting gene expression was compared to fed gene expression for both genotypes. Gene-based analysis confirmed prior findings, but provided no obvious alternative explanation for hypolipidemia. Pathway analysis of predicted upstream regulators showed modest but significant differences in the fed state, with a transcriptional signature of reduced Endoplasmic Reticulum (ER) stress (upstream regulators *Xbp1*, its processing enzyme *Ern1* (*Ire1*), and *Atf6*), and altered signatures of several cytokines including *IL6* (reduced), *IL21*, interferons gamma and alpha 2 (increased) (Figure 5, Table S2). More significant differences were seen after 16-h fasting, when a transcriptional profile consistent with the reduced activity of *Rictor*, the defining component of mTORC2, was seen with high significance, while patterns associated with *Myc* and *Mycn* were upregulated. The large preponderance of changes observed between fed and fasting states in wild-type mice, including strong upregulation of PPARα-responsive genes, was however conserved in heterozygous animals (Figure 5, Table S2).

In view of the transcriptomic evidence of reduced Rictor activity, plasma and hepatic amino acid and β-hydroxybutyrate concentrations were determined. This revealed increased β-hydroxybutyrate in the liver from fed *Pik3r1*^*WT/Y657*∗^ mice with a trend towards an increase in plasma, but no difference between genotypes when fasted (Figure S7A). The fall in plasma amino acid concentrations seen in fasting wild-type mice was lost in *Pik3r1*^*WT/Y657*∗^ mice, with a trend towards a generalised increase in liver amino acids (Figure S7B,C). These findings add to the evidence for abnormal transitions between fed and fasted states in *Pik3r1*^*WT/Y657*∗^ mice, and suggest that reduced intra-hepatocyte amino acid concentrations on fasting do not explain the pronounced reduction in Rictor signalling suggested by transcriptomics.

## 4 Discussion

Most monogenic defects causing severe IR primarily affect adipose tissue development, and metabolically phenocopy common IR [1]. In contrast, humans with severe IR due to proximal insulin signalling defects do not have the dyslipidaemia, fatty liver, nor decreased plasma adiponectin concentration usual in common IR [6,7,13,36]. This was first noted in people with insulin receptor mutations [13], but more recently, has been described in SHORT syndrome [6,7], in which the lack of fatty liver and dyslipidaemia is particularly striking given concomitant lipodystrophy. Fatty liver and atherogenic dyslipidaemia are major sources of IR-related morbidity, so investigating how these are dissociated from IR in the face of proximal insulin signalling defects will potentially reveal novel approaches to mitigate IR-associated disease.

The novel mice generated, heterozygous for the Pik3r1 Y657∗ allele, show reduced growth, severe IR, and severely reduced adipose accretion on high-fat feeding. Although IR in *Pik3r1*^*R649W/WT*^ mice described previously was reported to spare skeletal muscle [31], we found skeletal muscle to be insulin resistant both in clamps, and *ex vivo,* in keeping with the ubiquitous expression of the mutant allele.

Despite severe IR, *Pik3r1*^*WT/Y657*∗^ mice were hypolipidemic on chow or HFD, and in fed and fasted states, while the increase in liver triglyceride usual in lipodystrophic IR was absent, validating it as a model of the human phenomenon we aimed to study. Knockout evidence that PI3K signalling mediates insulin's stimulation of liver *de novo* lipogenesis led us to expect reduced transcript levels of *Srebp* and its target genes (e.g. [16,17]), however we found no evidence supporting this or other candidate transcriptional mechanisms including reduced lipogenic activity of *Xbp1* [34].

RNA sequencing showed most insulin-responsive genes to respond normally to fasting and refeeding. Given elevated plasma insulin concentrations, this could be construed as showing compensated hepatic IR, as expected from a proximal insulin signalling defect. Fasting-responsive programmes, such as those orchestrated by PPARα, were intact in *Pik3r1*^*WT/Y657*∗^ mice, however pathway analysis suggested reduced ER stress signalling and showed altered inflammatory signatures in the fed state, with a strikingly reduced Rictor signature in the fasted state. These transcriptional clues may be pertinent to the protection from dyslipidaemia as ER stress signalling promotes fatty liver and dyslipidaemia [37], while Rictor drives hepatic lipogenesis [38], however further study is needed. Impaired induction of Rictor activity in this case may reflect Pik3r1's role in amino acid activation of Rictor [39].

*Pik3r1*^*R649W/WT*^ mice have been shown to have less subcutaneous adipose tissue [31] and reduced adipose expansion in obesogenic conditions [40], as in the current report, which was interpreted as evidence of lipodystrophy. However, constrained adipose expansion produces inflammation of overloaded, hypertrophic adipose tissue during chronic positive energy balance [1], and the normal adipocyte appearance, lack of adipose inflammation, and normal differentiation of preadipocytes *ex vivo* in both studies argue against this [31].

Reduced adipose mass can reflect altered energy balance rather than lipodystrophy. Reduction of PI3K pathway activity by overexpression of the lipid phosphatase *Pten* in mice was previously reported to enhance energy expenditure via brown adipose tissue activation [41], albeit without IR, while pharmacological or genetic inhibition of the p110α catalytic subunit of PI3K has also been associated with increased energy expenditure [[40], [41], [42], [43]]. Moreover, a similar energetic phenotype on brown adipose-specific knockout of Pik3r1 has recently been described [44]. On HFD we found that food intake and energy expenditure were both increased in *Pik3r1*^*WT/Y657*∗^ mice. Given unaltered locomotor activity, intestinal lipid absorption, and lean tissue growth, reduced adipose accumulation shows that increased energy expenditure outweighed increased food intake, and is thus the primary abnormality. Reduced thermal insulation in smaller animals is an unlikely explanation [43]; hence, we conclude that increased energy expenditure explains reduced adipose accretion. Increased brown adipose tissue activity is the most likely explanation, as in models with p110α inhibition [[40], [41], [42], [43]], but this requires further study.

Increasing brown adipose tissue function reduces plasma triglyceride and cholesterol concentrations [45,46], and this reduces atherogenesis when liver scavenging of triglyceride-depleted lipoproteins is intact [45]. This accords with the hypolipidemia of *Pik3r1*^*WT/Y657*∗^ mice, and humans with SHORT syndrome. As brown fat is now known to occur in humans, it would be of interest to establish whether it is hyperactive in people with SHORT syndrome. In addition, given the normal lipid profile despite severe IR in people with insulin receptor mutations [13], and the reduced adipose tissue in mice harbouring a common dominant negative insulin receptor allele on an obesogenic diet [47], it will be of interest to assess whether the bioenergetic consequences of reduced PI3K signalling are also seen in the face of insulin receptor dysfunction.

## 5 Conclusions

We report a novel mouse model of SHORT syndrome recapitulating the severe IR without dyslipidaemia seen in humans. Hepatic lipogenesis and lipogenic gene expression were unimpaired, and transcriptomic pathway analysis suggested only mild nutritional state-specific abnormalities in liver gene expression. White adipose tissue was reduced on HFD, but did not show inflammation of hypertrophy, while energy expenditure was increased, raising the testable possibility that the reduced adipose mass in SHORT syndrome does not represent true “lipodystrophy”. The most likely mechanism explaining uncoupling of IR from dyslipidaemia in SHORT syndrome is increased brown adipose activity, consistent with other murine evidence that inhibition of PI3K results in beneficial metabolic alterations as well as IR.

## Author contributions

Conceptualization, RKS, AK; Methodology, SV, AK, PV, IZ, JJ, JG, JW, RA Formal Analysis, AK, IZ, SV, IHD, PT, DAB, DH, RK, PV, JG, JW, JJ, RKS; Investigation, AK, IZ, SV, IL, IHD, PT, DAB, DH, RK, PV, JJ, RKS; Writing – Original Draft, RKS, AK, IHD; Writing – Review & Editing, IZ, SV, PT, DAB, DH, RK, AVP, PV, JJ, SO; Supervision, RKS; Project Administration, RKS; Funding Acquisition, RKS, SO, AVP.
