# Supplementary material for: Truncation of Pik3r1 causes severe insulin resistance uncoupled from obesity and dyslipidaemia by increased energy expenditure
Source: Mol Metab. 2020 May 19;40:101020. doi: 10.1016/j.molmet.2020.101020 (PMC7385515; doi:10.1016/j.molmet.2020.101020)
Supplement: Multimedia component 1 [file mmc1.docx]

**Supplementary Information for**

**Truncation of Pik3r1 causes severe insulin resistance uncoupled from obesity and dyslipidemia by increased energy expenditure**

Albert Kwok, Ilona Zvetkova, Sam Virtue, Ineke Luijten, Isabel Huang-Doran, Patsy Tomlinson, David A. Bulger, James West, Steven Murfitt, Julian Griffin, Rafeah Alam, Daniel Hart, Rachel Knox, Peter Voshol, Antonio Vidal-Puig, Jørgen Jensen, Stephen O’Rahilly, Robert K Semple

Correspondence: Robert K. Semple; rsemple@ed.ac.uk

**This PDF file includes:**

Tables S1 to S4

Figs. S1 to S7

**Table S1. Fasting biochemical profile of blood from female Pik3r1^WT/WT^ and Pik3r1^WT/Y657*^ mice.** Results are expressed as mean ± S.E.M. * denotes values differing significantly (P < 0.05) between genotypes. Statistical comparisons were undertaken using the student’s t-test.

|  | **Pik3r1^WT/WT^ (n=10)** | **Pik3r1^WT/Y657*^ (n=14)** | **p** |
| --- | --- | --- | --- |
| **Glucose (mmol/L)** | 11.6 ± 1.0 | 10.9 ± 1.0 | 0.62 |
| **Insulin (pmol/L)** | 172.5 ± 43.1 | 241.5 ± 51.6 | 0.27 |
| **Leptin (μg/L)** | 2.7 ± 0.6 | 2.5 ± 0.6 | 0.71 |
| **Adiponectin (mg/L)** | 41.9 ± 1.5 | 40.1 ± 1.6 | 0.41 |
| **Total cholesterol (mmol/L)** | 2.8 ± 0.1* | 2.4 ± 0.1* | 0.01 |
| **HDL cholesterol (mmol/L)** | 1.3 ± 0.0* | 1.0 ± 0.1* | 0.01 |
| **Triglycerides (mmol/L)** | 1.3 ± 0.1* | 1.1 ± 0.1* | 0.02 |
| **Testosterone (μg/L)** | 0.2 ± 0.1 | 0.2 ± 0.0 | 0.51 |

**Table S2. Pathway Analysis of Liver Transcriptome: Upstream regulators predicted to show differential activity**

| **Upstream Regulator** | **Comment** | **Activation z-score** | **p-value of overlap** |
| --- | --- | --- | --- |
| **Pik3r1^WT/Y657*^ *vs* wild type (fed)** | | | |
| STAT1 | Growth factor/cytokine-activated transcription factor | 2.0 | 1.0E-07 |
| IL21 | Interleukin 21 | 2.1 | 1.6E-08 |
| IFNG | Interferon γ | 2.2 | 9.3E-10 |
| UDP-D-glucose | Intermediary in glycogen synthesis | 2.2 | 3.5E-08 |
| mir-223 |  | 2.3 | 5.8E-06 |
| IFNA2 | Interferon α2 | 2.3 | 1.8E-08 |
| tretinoin | All-*trans* retinoic acid (ATRA) | 2.6 | 4.5E-08 |
| APP | Amyloid beta precursor protein | 3.7 | 3.7E-09 |
| XBP1 | Transcriptional mediator of ER stress response | -3.7 | 5.1E-08 |
| ERN1 | Ire1; endocribonuclease that splices Xbp1 mRNA to active form in response to ER stress | -3.3 | 4.0E-10 |
| PTGER4 | Prostaglandin E receptor 4 | -3.2 | 7.7E-07 |
| tunicamycin | chemical - endogenous non-mammalian | -3.0 | 3.6E-07 |
| ATF6 | ER stress sensor and initiator of ER stress response | -2.6 | 7.1E-06 |
| KRAS |  | -2.5 | 3.0E-06 |
| POR | Cytochrome p450 oxidoreductase; important for ATRA metabolism | -2.4 | 8.7E-07 |
| IL6 | Interleukin 6 | -2.0 | 2.4E-13 |
| 1,2-dithiol-3-thione | Chemical anti-oxidant | -2.0 | 8.2E-11 |
| **Pik3r1^WT/Y657*^ *vs* wild type (fasted)** | | | |
| MYCN | Transcription factor | 5.5 | 6.7E-24 |
| methylprednisolone | Glucocorticoid | 5.1 | 3.2E-20 |
| MYC | Transcription factor | 3.4 | 2.5E-16 |
| ciprofibrate | PPARα agonist | 2.5 | 3.1E-11 |
| 1,2-dithiol-3-thione | Chemical anti-oxidant | 3.6 | 9.1E-08 |
| nitrofurantoin | Chemical anti-microbial | 3.2 | 6.7E-07 |
| NKX2-3 | Homeobox gene | 3.1 | 1.5E-06 |
| TRIM24 | Nuclear hormone receptor accessory protein | 4.1 | 8.4E-06 |
| guanidinopropionic acid | Creatine monohydrate analogue | 2.0 | 9.4E-06 |
| RICTOR | Core component of mTORC2 | -8.6 | 2.3E-36 |
| sirolimus | mTORC1 inhibitor | -4.3 | 2.4E-17 |
| ACOX1 | Acyl-coenzyme A oxidase 1 | -3.5 | 3.9E-14 |
| 5-fluorouracil | A fluorinated pyrimidine | -3.2 | 2.1E-13 |
| HNF4A | Hepatic nuclear factor 4α | -3.2 | 1.0E-11 |
| ST1926 | Synthetic retinoid | -3.8 | 7.5E-10 |
| tretinoin | All-*trans* retinoic acid (ATRA) | -2.1 | 1.8E-08 |
| CD 437 | Toxin; DNA polymerase α inhibitor | -3.2 | 2.0E-08 |
| PPARA | PPARα | -2.7 | 5.3E-08 |
| TP53 | Tumour suppressopr p53 | -2.8 | 1.1E-07 |
| HNF1A | Hepatic nuclear factor 1α | -2.4 | 1.1E-06 |
| PPARG | PPARγ | -3.3 | 7.1E-06 |
| IFNA2 | Interleukin α2 | -2.6 | 9.5E-06 |
| **Fasted vs Fed (wild type)** | | | |
| PPARA | PPARα; nuclear hormone mediating metabolic response to fasting | 8.6 | 9.6E-39 |
| pirinixic acid | PPARα agonist | 6.3 | 3.9E-43 |
| clofibrate | PPARα agonist | 5.6 | 8.8E-18 |
| fenofibrate | PPARα agonist | 5.3 | 5.5E-20 |
| ciprofibrate | PPARα agonist | 4.9 | 1.0E-28 |
| rosiglitazone | PPARγ agonist | 4.3 | 2.0E-07 |
| sirolimus | mTORC1 inhibitor | 4.2 | 1.8E-08 |
| bezafibrate | PPARα agonist | 4.1 | 1.2E-18 |
| NPPB | Natriuretic peptide B | 4.1 | 4.1E-06 |
| AHR | Aryl hydrocarbon receptor | 3.8 | 8.0E-08 |
| bromobenzene |  | 3.8 | 2.2E-07 |
| POR | Cytochrome p450 oxidoreductase | 3.8 | 2.0E-28 |
| di(2-ethylhexyl) phthalate |  | 3.8 | 5.1E-07 |
| PPARD | PPARδ | 3.4 | 6.1E-13 |
| INSIG2 | Insulin-induced gene 2 | 3.5 | 4.7E-07 |
| PNPLA2 | Patatin-like phospholipase domain-containing 2 | 3.2 | 1.1E-10 |
| INSIG1 | Insulin-induced gene 1 | 3.2 | 1.6E-11 |
| gemfibrozil | PPARα agonist | 3.2 | 5.5E-07 |
| camptothecin | DNA topoisomerase inhibitor | 3.1 | 3.7E-06 |
| GW7647 | PPARα agonist | 3.0 | 3.5E-07 |
| troglitazone | PPARγ agonist | 2.9 | 2.6E-10 |
| PPARG | PPARγ agonist | 2.9 | 1.6E-14 |
| KLF15 | Krüppel-like factor 15; increased by glucocorticoids; suppressed by PI3K | 2.9 | 2.0E-08 |
| EP300 | Histone acetyltransferase p300 | 2.7 | 1.1E-06 |
| SIRT6 | Sirtuin 6 | 2.5 | 7.7E-06 |
| DGAT1 | Diacylglycerol transferase-1 | 2.5 | 2.0E-06 |
| cholesterol |  | 2.4 | 1.3E-09 |
| miR-16-5p |  | 2.4 | 9.3E-06 |
| palmitic acid |  | 2.4 | 5.7E-07 |
| SIRT1 | Sirtuin 1 | 2.2 | 1.6E-06 |
| PTEN | Phospholipid phosphatase antagonising PI3K | 2.2 | 5.1E-07 |
| benzo(a)pyrene |  | 2.1 | 6.0E-06 |
| bexarotene |  | 2.0 | 4.1E-08 |
| TMBIM6 | Bax inhibitor | -2.1 | 3.8E-07 |
| ATF4 | Transcription factor | -2.3 | 1.1E-06 |
| MYCN | Transcription factor | -2.3 | 3.2E-12 |
| ATF6 | ER stress sensor and initiator of ER stress response | -2.5 | 2.6E-08 |
| thapsigargin | Sarcoplasmic Ca^2+^ channel inhibitor; blocks later stages of autophagy | -2.6 | 1.6E-07 |
| lipopolysaccharide |  | -2.7 | 1.9E-07 |
| FBXO32 | F-box only protein 32 | -2.7 | 1.6E-09 |
| RAB1B | Small GTP-binding protein | -2.8 | 4.4E-06 |
| L-dopa |  | -2.9 | 5.2E-07 |
| methylprednisolone | Glucocorticoid | -3.0 | 7.0E-25 |
| HSD17B4 | Enzyme involved in peroxisomal fatty acid β oxidation | -3.0 | 3.5E-07 |
| Insulin |  | -3.1 | 1.2E-09 |
| ESR1 | Ligand-dependent nuclear receptor | -3.2 | 6.7E-09 |
| TNF | Tumour necrosis factor α | -3.2 | 8.3E-09 |
| HNF1A | Transcription regulator | -3.2 | 8.4E-13 |
| EHHADH | Enzyme involved in peroxisomal fatty acid β oxidation | -3.2 | 7.9E-10 |
| isoquercitrin | Plant-derived flavenoid | -3.2 | 8.0E-10 |
| ATP7B | Copper-transporting ATPase | -3.3 | 1.7E-07 |
| ezetimibe | Inhibitor of intestinal cholesterol uptake | -3.3 | 5.5E-09 |
| gentamicin | Antimicrobial | -3.9 | 2.0E-09 |
| MYC | Transcription factor | -4.0 | 2.5E-16 |
| SREBF1 | Lipogenic transcription factor | -4.0 | 8.6E-12 |
| ACOX1 | Acyl-coenzyme A oxidase 1 | -4.1 | 2.4E-29 |
| SREBF2 | Cholesterologenic transcription factor | -4.2 | 9.9E-20 |
| mibolerone | Synthetic androgen | -4.5 | 1.8E-13 |
| SCAP | Sterol regulatory element-binding protein cleavage-activating protein | -4.8 | 6.1E-17 |
| ERN1 | Ire1; endocribonuclease that splices Xbp1 mRNA to active form in response to ER stress | -5.3 | 1.8E-17 |
| tunicamycin | Blocks N-linked glycosylation; commonly used to provoke ER stress/unfolded protein response | -5.7 | 4.2E-24 |
| XBP1 | Transcriptional mediator of ER stress response | -8.2 | 4.7E-34 |
| **Fasted vs Fed (***Pik3r1^WT/Y657*^***)** | | | |
| PPARA | PPARα | 7.6 | 2.3E-35 |
| TRIM24 | Nuclear hormone receptor accessory protein | 5.8 | 4.7E-07 |
| pirinixic acid | PPARα agonist | 5.4 | 8.7E-32 |
| ciprofibrate | PPARα agonist | 5.4 | 2.0E-13 |
| clofibrate | PPARα agonist | 5.0 | 1.2E-13 |
| INSIG1 | Insulin-induced gene 1 | 4.7 | 2.2E-09 |
| POR | Cytochrome p450 oxidoreductase; important for ATRA metabolism | 3.8 | 3.3E-28 |
| NPPB | Natriuretic peptide B | 3.6 | 5.9E-06 |
| miR-124-3p |  | 3.4 | 8.9E-07 |
| INSIG2 | Insulin-induced gene 1 | 3.4 | 6.3E-07 |
| MYCN | Transcription factor | 3.4 | 3.5E-13 |
| bezafibrate | PPARα agonist | 3.3 | 1.4E-16 |
| fenofibrate | PPARα agonist | 3.3 | 1.1E-14 |
| PNPLA2 | Patatin-like phospholipase domain-containing 2 | 3.3 | 2.4E-06 |
| NR3C1 | Ligand-dependent nuclear receptor | 2.9 | 6.2E-09 |
| dexamethasone | Glucocorticoid | 2.7 | 1.0E-16 |
| SLC13A1 | Transporter | 2.7 | 2.9E-11 |
| KLF15 | Krüppel-like factor 15; increased by glucocorticoids; suppressed by PI3K | 2.6 | 9.3E-10 |
| arsenic trioxide |  | 2.6 | 5.9E-06 |
| PPARGC1A | Nuclear hormone receptor co-activator | 2.5 | 2.7E-10 |
| sterol |  | 2.5 | 2.2E-07 |
| FGF19 | Fibroblast growth factor 19 | 2.4 | 5.4E-06 |
| GW7647 | PPARα agonist | 2.4 | 3.9E-06 |
| palmitic acid |  | 2.2 | 3.1E-06 |
| rosiglitazone | PPARγ agonist | 2.1 | 7.9E-08 |
| KRAS |  | 2.1 | 2.6E-06 |
| prednisolone | Glucocorticoid | 2.1 | 5.3E-06 |
| dihydrotestosterone |  | -2.1 | 3.1E-11 |
| tretinoin | All-*trans* retinoic acid (ATRA) | -2.2 | 5.3E-08 |
| EHHADH | Enzyme involved in peroxisomal fatty acid β oxidation | -2.3 | 6.3E-07 |
| 1,4-bis[2-(3,5-dichloropyridyloxy)]benzene | Potent p450 cytochrome inducing xenobiotic | -2.4 | 2.1E-06 |
| ERBB2 | Human epidermal growth factor receptor 2 | -2.5 | 2.6E-10 |
| TO-901317 | Liver X receptor agonist | -2.8 | 2.1E-15 |
| OSM | Cytokine influencing expression of many other cytokines | -2.8 | 1.7E-06 |
| HNF1A | Hepatic nuclear factor 1α | -2.9 | 9.4E-10 |
| PCK1 | PEPCK; gluconeogenic enzyme | -3.0 | 3.0E-06 |
| ATP7B | Copper-transporting ATPase | -3.0 | 1.0E-09 |
| TGFB1 | Transforming growth factor β | -3.0 | 8.2E-10 |
| APP | Amyloid beta precursor protein | -3.2 | 8.7E-12 |
| MLXIPL | Carbohydrate response element-binding protein | -3.2 | 9.8E-07 |
| isoquercitrin | Plant-derived flavenoid | -3.2 | 1.1E-09 |
| E2F1 | Retinoblastoma-target transcription factor | -3.3 | 1.0E-06 |
| Ins1 | Insulin | -3.5 | 2.7E-08 |
| ezetimibe | Chemical drug | -3.6 | 1.2E-07 |
| gentamicin | Antimicrobial | -3.6 | 7.2E-09 |
| mibolerone | Synthetic androgen | -3.9 | 1.8E-07 |
| TNF | Tumour necrosis factor α | -4.0 | 5.9E-09 |
| ESR1 | Ligand-dependent nuclear receptor | -4.0 | 4.7E-09 |
| ANGPT2 | Angiopoietin 2 | -4.2 | 7.1E-07 |
| ACOX1 | Acyl-coenzyme A oxidase 1 | -4.3 | 4.2E-18 |
| SREBF2 | Cholesterologenic transcription factor | -4.5 | 2.7E-18 |
| ERN1 | Ire1; endocribonuclease that splices Xbp1 mRNA to active form in response to ER stress | -4.8 | 9.4E-11 |
| SREBF1 | Lipogenic transcription factor | -4.9 | 1.3E-15 |
| tunicamycin | Blocks N-linked glycosylation; commonly used to provoke ER stress/unfolded protein response | -5.1 | 2.8E-18 |
| SCAP | Sterol regulatory element-binding protein cleavage-activating protein | -5.3 | 1.5E-15 |
| RICTOR | Core component of mTORC2 | -6.1 | 4.6E-10 |
| XBP1 | Transcriptional mediator of ER stress response | -7.6 | 5.7E-22 |

**Table S3. Details of assays used to measure plasma parameters.** All the measurements were conducted following the manufacturer’s instructions.

| **Plasma parameter** | **Assay manufacturer** |
| --- | --- |
| Glucose | Siemens Healthcare Diagnostics |
| Insulin | Meso Scale Discovery or Crystal chem |
| Leptin | Meso Scale Discovery |
| Adiponectin | Meso Scale Discovery |
| Triacylglycerol | Siemens Healthcare Diagnostics |
| Total cholesterol | Siemens Healthcare Diagnostics |
| HDL cholesterol | Randox |
| VLDL cholesterol | Cusabio |
| Free fatty acids | Roche |
| Testosterone | IBL international |

**Table S4. Reagents used for Gene Expression Analysis.**

| **ANTIBODIES** | | |
| --- | --- | --- |
| **Target** | **Commercial Supplier** | **Catalogue number** |
| p85α | Cell signalling | 4257S |
| p85β | Novus | NBP2-19817 |
| p110α | Cell signalling | 4249S |
| p110β | Cell signalling | 3011S |
| pAkt^S473^ | Cell signalling | 9271S |
| pAkt^T308^ | Cell signalling | 9275S |
| Total Akt | Cell signalling | 2920S |
| β-actin | Cell signalling | 4967S |
| GAPDH | Abcam | ab8245 |
|  | | |
| **REAL TIME QUANTITATIVE PCR** | | |
| **Target** | **Primers** | **Probe** |
| Pck1 | For: TGTGGGCGATGACATTGC  Rev: TGGCATTTGGATTTGTCTTCAC | FAM- TATCAACCCAGAAAACGGGTTTTTTG-TAMRA |
| G6pc | For: CCAACCACAAGATGACGTTCA  Rev: ACTCTTGCTATCTTTCGAGGAAAGA |  |
| Srebp1c | For: GCCATGGATTGCACATTTGA  Rev: GGCCCGGGAAGTCACTG | FAM-GACATGCTCCAGCTCATCAACAACCAAG-TAMRA |
| Acc1 | For: AGCATGTCTGGCTTGCACCTAGTA  Rev: CTGCTGCAATACCATTGTTGGCGA | N/A |
| Fasn | For: GCCCAGACAGAGAAGAGGCA  Rev: CTGACTCGGGCAACTTCCC | FAM-GGAGGAGGTGGTGATAGCCGGTATGTC-TAMRA |
| Scd1 | For: CTTGCGGATCTTCCTTATCATT  Rev: GATCTCGGGCCCATTCG | FAM-ACCATGGCGTTCCAGAATGACGTGT-TAMRA |
| Mvk | For: TCCAGCAAGGGACGATGTC  Rev: CTTGGTGTTGGTGAGCAGGAT | N/A |
| B2m | For: ACTGATACATACGCCTGCAGAGTT  Rev: TCACATGTCTCGATCCCAGTAGA | N/A |
| Eef1a1 | For: CACATCCCAGGCTGACTGT  Rev: TCGGTGGAATCCATTTTGTT | FAMCCTGATTGTTGCTGCTGGTGTTGGTGA-TAMRA |
| Ppia | For: TTCCTCCTTTCACAGAATTATTCCA  Rev: CCGCCAGTGCCATTATGG | FAM-ATTCATGTGCCAGGGTGGTGACTTTACAC-TAMRA |
|  | **Thermo Fisher Scientific Assay ID** | |
| *Gck* | Mm00439129_m1 | |
| *Igfbp1* | Mm00515154_m1 | |
| *Srebp2* | Mm 01306292_m1 | |
| *Nsdhl* | Mm 00477897_m1 | |
| *Ywhaz* | Mm 03950126_s1 | |
| *Pklr* | Mm00443090_m1 | |
| *Acacb* | Mm01204671_m1 | |
| *Dgat2* | Mm00499536_m1 | |

**Figure S1. Generation of Pik3R1^WT/Y657*^ mice.** (A) Targeting vector used to generate WT/657* mice. (B) Protein expression of class 1A PI3K subunits in liver, skeletal muscle (Quadriceps), inguinal and epididymal white adipose tissues. The loading control for liver and adipose tissue lysates was β-actin while GAPDH was used as a loading control for skeletal muscles. Coomassie stained gels indicate even loading of lysates. Arrows indicate the p85β protein bands.

**Figure S2. Effect of Y657* on embryonic development.** (A)-(B) Representative images and weights (adjacent scatter plot) of Pik3r1^WT/WT^ (WT/WT), Pik3r1^WT/Y657*^ (WT/Y657*) and Pik3r1^Y657*/Y657*^ (Y657*/Y657*) embryos at E11.5. Scale bar = 5mm. (C)-(E) Embryo weights at (C) E13.5 (n = 22 for WT/WT and 19 for WT/Y657*), (D) E15.5 (n=17 and 13) and (E) E18.5 (n=18 and 22). Data are presented as mean ± SD. * = p < 0.05; ** = p < 0.01; ***=p<0.001.

**Figure S3. Body composition change and adipokine concentrations normalised to total fat mass on high fat diet.** (A) % Gain in fat and lean mass on chow (n=17 for Pik3r1^WT/WT^ (WT/WT) and 12 for Pik3r1^WT/Y657*^ (WT/Y657*)) (B) % Gain in fat and lean mass on 45% fat diet (n=17 and 12). Gain in mass is shown as week 16 fat or lean mass expressed as a percentage of corresponding week 8 values. Data are presented as mean ± SD; *** = p < 0.001. (C) Plasma leptin concentration (μg/L) normalised to TD-NMR-derived absolute fat mass (g) (n=15 for WT/WT and n=11 for WT/Y657*). (D) Plasma adiponectin concentration (mg/L) similarly normalised to absolute fat mass (n=16 and 11). Data are presented as mean ± SD. * = p < 0.05 and *** = p < 0.001.

**Figure S4. Glucose homeostasis in female Pik3r1^WT/Y657*^** **mice on chow.** (A) Oral glucose tolerance testing and area under the curve (AUC) comparison (n=10 for Pik3r1^WT/WT^ (WT/WT) and n=15 for Pik3r1^WT/Y657*^ (WT/Y657*)) (B) Blood insulin concentrations during OGTT and corresponding AUCs (C) Insulin tolerance testing and AUCs (n=10 and 14). Data are presented as mean ± SD. * = p < 0.05 and ** = p < 0.01.

**Figure S5. Insulin-induced Akt Thr308 phosphorylation in insulin target tissues of Pik3r1^WT/Y657*^ mice.** (A)-(D) 16 week-old male mice were injected intraperitoneally with 2U/kg insulin and tissues were harvested 10 minutes later. Representative immunoblots for pAkt^T308^ and total Akt and corresponding quantification are shown for 6 animals per genotype per condition for (A) liver (B) skeletal muscle (Quadriceps) (C) eWAT and (D) ingWAT. (E) Representative immunoblots and quantification for Soleus and EDL lysates after *ex vivo* insulin stimulation (n=5 and 4). Numerical data are presented as mean ± SD. * = p < 0.05, ** = p < 0.01, ***p = < 0.001 and **** = p < 0.0001.

**Figure S6. Selected liver transcriptional responses to fasting and refeeding of Pik3r1^WT/Y657*^ mice.** mRNA expression was determined by real time quantitative PCR for (A) *Pklr* (B) *Acacb*, (C) *Dgat2*, (D) *G6pc*, and (E) *Gck.*  (G) *G6pc*:*Gck* expression ratio at the same time points. Data are presented as mean ± SD * = p < 0.05; ** = p < 0.01.

**Figure S7. Plasma and liver amino acid and beta-hydroxybutyrate profiles of fed and fasted mice.** (A) β-hydroxybutyrate concentrations in liver and plasma of male Pik3r1^WT/WT^ (WT/WT) and Pik3r1^WT/Y657*^(WT/Y657*) mice (n=6). Means ± SD are shown. * = p < 0.05. (B) Volcano plots showing differences in plasma amino acid concentrations between nutritional states (outside plots) and genotype (central plots). (C) Volcano plots showing differences in liver amino acid concentrations between nutritional states (outside plots) and genotype (central plots). Selected amino acids are indicated by single letter codes. 6 mice were studied in all groups.
